# Informed consent in medical research

**DOI:** 10.1054/bjoc.2001.2135

**Published:** 2001-11

**Authors:** E G DeRenzo

**Affiliations:** Center for Ethics, Washington Hospital Center, Washington, DC, USA


*British Journal of Cancer* (2001) **85**(11), 1811–1812
© 2001 Cancer Research Campaign
doi: 10.1054/ bjoc.2001.2135, available online at http://www.idealibrary.com on IDEAL
http://www.bjcancer.com

Review

### Informed consent in medical research

**L Doyal and JS Tobias**

BMJ Books: London, November 2000 pp 336 ISBN 0 7279 1486 Price: £50.00

*Informed Consent in Medical Research* by Doyal and Tobias is both a fascinating and frustrating book. Several of the ways in which it is fascinating relate to the reason the book was written, the book's organization, and the wealth of opinion presented. The frustrations engendered in the reading of the book spring from the same sources.

The book was written to highlight, examine, and extend discussion around a single question, 'Should the *BMJ* publish reports of research in which informed consent of the subjects has not been obtained?' The question, brought into focus by internal *BMJ* debate over whether or not to publish two particular studies (see Part 2, Chapters 7, 8, and 9), is an important one – not only for the *BMJ* but for the world of research publications, in general. That Richard Smith, *BMJ* Editor, acknowledges explicitly in the book's Foreword that the internal debate, and the decision to produce the book, resulted from uncertainty and confusion, is to his credit and to that of the *BMJ* staff. To suggest that there might be, at this stage in the evolution of research ethics, universal certitude about whether there are appropriate conditions under which it would be ethically acceptable to waive informed consent would be to admit to a frightening lack of insight about the ethical intricacies of clinical research. Rather, a healthy uncertainty and confusion about such refined ethical points as consent waivers, and as addressed in Part 4, Chapter 25, just what does 'fully' mean in the term-of-art of 'fully informed consent' builds confidence that there is a vigorous and thoughtful consideration of the wide range of ethical issues inherent in the publication of biomedical research at the *BMJ* and its sister publications.

The frustration here is that the question never gets answered. Now, just having applauded the *BMJ* for publicly wallowing in their confusions and uncertainties, it may seem odd to suggest that the book ought to have settled the matter. And I am not suggesting that the matter can be settled for all time. But one might have hoped for an epilogue, or the like, indicating whether or not all the backing and forthing had convinced the *BMJ* editors and staff that they should stand by their original decision, or that now, after all this debate, they ought to set a policy only to publish where consent has been obtained. This frustration comes from this author's perspective as a clinical research ethicist. Unlike the philosopher whose calling it is to debate indefinitely (and no doubt the book's core question, and the innumerable sub-questions it spawns, could be debated profitably, indefinitely), the clinical ethicist is a practical sort, called on to assist in coming to the most ethically justifiable solutions at the time solutions need to be found. But then one can just as easily debate timing, and perhaps the time to write the *BMJ* policy has not yet come.

At the heart of what I am categorizing as the second primary source of the book's blessings and curses is all the backing and forthing. That is, the book has a most interesting yet exhausting organization. Part 1 starts out straightforwardly enough, providing the texts of the Nuremberg Code and the Declaration of Helsinki (albeit the most recent past version of Helsinki, an obsolescence in a brand new book that ought to give us all pause about the certitude with which we think we can settle these ethical issues once and for all). Absolutely riveting is Chapter 2 about the *BMJ*'s correspondent at the Nuremberg trial, as are all the Chapters in this first section, with the snippets from Pappworth and Beecher well placed. Part 2 also opens in a tidy fashion, presenting the issue(s), the stimulus papers, and then laying out the debate's polarities. From there on out, however, the book becomes a mass of orchestrated chaos. Truly, it is like a Cage symphony, with every note placed with precision, but placed in such a way that as each part of the orchestra talks, another part answers, and then another part pipes up, and then another cuts in, and on and on to total intellectual exhaustion. For the research ethics groupie, the organization may be Nirvana. For the less research ethics sophisticated, it may create a bit of a blur. But, like any fine symphony, the final movement of the score brings even the weariest listener back into its grasp. The book's editors achieve their grand finale grandly. Part 5's chapters tie up the work beautifully.

The final comment about the book's benefits and burdens is, again, reminiscent of all the various instruments in the orchestra. The most striking feature of the book is its richness and breadth. Here, unlike a Cage symphony that can sound sparsely notated and pared down, one arrives at the end of the book and feels bathed in the expansiveness of Wagnerian opera. The book presents so many voices.

Great attention was paid to assuring that a wide variety of persons and perspectives were heard. This is one of the book's many joys. But the burden of goals of inclusiveness is that the attempt is often sisyphean. No matter how many times one pushes up that rock of diversity, some voices are either not lifted up or drowned out because of disparities in proportions. Here, the largest number of voices is professional, whose analyses of the consent question tend to cluster around consequentially grounded arguments or come out of duty-based thinking. Few address the problem from a virtue ethics perspective. If, for example, the virtue-based arguments had been made more loudly, some of the authors, who, despite their own best efforts, continue to inappropriately conflate the goals of treatment with the goals of research, might have been shown to be mounting arguments that do not hold up in the face of even more intensely focused debate. Additionally, it is interesting to note that in this vast sea of professional disagreement, the few lay authors (see Kulsum Winship's response in Part 2, Chapter 12 and Chapter 28 in Part 4) are consistent in their call for conducting research only with properly obtained (i.e., parental and/or surrogate included) consent. If more voices had been heard from those who have been research subjects, and their views had been predominantly on the must-have-consent side, the scales may have tipped.

As it is, we are left continuing to wallow in the *BMJ*'s and our own uncertainties and confusions. And while this may be the best possible place to be right now, it is uncomfortable. Thus, perhaps legitimizing our discomforts while supporting and encouraging further debate and discussion about the need for consent of subjects in published research reports is, in the end, the greatest achievement of what is surely, as Richard Smith hoped readers would conclude, a marvellous book.

**E G DeRenzo**
*Bioethicist*
*Center for Ethics*
*Washington Hospital Center*
*Washington DC*
*USA*

*British Journal of Cancer* (2001) **85**(11), 1812
© 2001 Cancer Research Campaign
doi: 10.1054/ bjoc.2001.2198, available online at http://www.idealibrary.com on IDEAL

### Erratum

**Kirsten ras mutations in patients with colorectal cancer: the “RASCAL II” study**
HJN Andreyev et al.

*Br J Cancer* **85**(5): 692–696

The names of two of the authors of this paper were published incorrectly, and should have appeared as follows:

DT Croke (rather than CT Croke)
DR Smith (rather than R Smith)

The publishers wish to apologise for this error.

 *© 2001 Cancer Research Campaign*

